# The link between spirituality and longevity

**DOI:** 10.1007/s40520-023-02684-5

**Published:** 2024-02-11

**Authors:** Ligia J. Dominguez, Nicola Veronese, Mario Barbagallo

**Affiliations:** 1 School of Medicine, Kore University of Enna, 94100 Enna, Italy; 2 Geriatric Unit, Department of Medicine, University of Palermo, 90127 Palermo, Italy

**Keywords:** Spirituality, Longevity, Aging, Religiosity, Religion, Mortality, Well-being

## Abstract

We are facing an inverted demographic pyramid with continuously growing aged populations around the world. However, the advances that prolong physical life not always contemplate its psychological and social dimensions. Longevity is a complex outcome influenced by a wide range of factors, including genetics, lifestyle choices, access to healthcare, socio-economic conditions, and other environmental factors. These factors have been generally considered in the compelling research that seeks the determinants of longevity, particularly those concerning personal lifestyle choices, socioeconomic conditions, and molecular mechanisms proposed to mediate these effects. Nonetheless, fundamental aspects that can affect health and well-being, such as spirituality and religiosity, have been somehow left aside despite numerous epidemiological studies showing that higher levels of spirituality/religiosity are associated with lower risk of mortality, even after adjusting for relevant confounders. Because spirituality/religiosity are dimensions of great value for patients, overlooking them can leave them with feelings of neglect and lack of connection with the health system and with the clinicians in charge of their care. Integrating spirituality and religiosity assessment and intervention programs into clinical care can help each person obtain better and complete well-being and also allowing clinicians to achieve the highest standards of health with holistic, person-centered care. The present narrative review aims to explore the available evidence of a relationship between spirituality/religiosity and longevity and discusses the possible mechanisms that can help explain such relationship.

## Introduction

Old age is a season of life that has always questioned humans, entailing a complex phenomenon, made up of various elements and dimensions: biological, genetic, physical, psychological, and also cultural, social, and environmental. Because all of these elements find a different combination in each of us, getting old is a very personal fact, which does not allow easy generalizations. Times and ways of getting old have changed today: we are facing an inverted demographic pyramid with continuously growing aged populations around the world [1]. Improved living conditions and modern medicine have contributed to the global increase in life expectancy [2]. However, the advances that prolong physical life not always contemplate its psychological and social dimensions.

Spirituality and longevity are concepts that have been explored in numerous studies and discussions in relation to aging. While there is no definitive scientific evidence linking directly spirituality and longevity [3], some research suggests that spiritual practices and beliefs may have indirect effects on physical and mental health, which could potentially contribute to longer life spans. For example, there are meta-analyses indicating that some factors connected to spirituality such as purpose in life and life satisfaction are associated with 17% and 12% reduced mortality risk, respectively [4, 5]. Contrariwise, social isolation and loneliness have been associated with 29% and 26% increased mortality risk, respectively [6]. Religious service attendance was also associated with a lower risk of death from despair (related to drugs, alcohol, and suicide) among health care professionals [7]. Attending a religious service more than once per week was associated with 33% lower all-cause mortality in women compared with those who had never attended religious services [8].

Longevity, as well as aging, is a complex outcome influenced by a wide range of factors, including genetics, lifestyle choices, access to healthcare, socio-economic conditions, and other environmental factors [1]. In general, these factors have been taken into account in the compelling research that seeks the determinants of longevity, particularly those concerning personal lifestyle choices [9] and socioeconomic conditions [10]; various molecular mechanisms proposed to mediate these effects have been studied [11, 12]. However, fundamental aspects that can affect health and well-being, such as spirituality and religiosity, have been somehow left aside. This may be due, at least in part, to the methodological difficulties of performing this type of research related to the heterogeneity of definitions and evaluation tools of these dimensions and the even more difficult transferring of the results into practical applications [3]. In addition, while some evidence suggests that certain spiritual practices and beliefs may indirectly contribute to better health and well-being, it is essential to acknowledge that causation is challenging to establish in this context. It is also crucial to keep in mind that the relationship between spirituality and longevity is complex and multifaceted, and individual experiences may largely and significantly vary.

The present narrative review aims to explore the available evidence of a relationship between spirituality/religiosity and longevity and discusses the possible mechanisms that can help explain such relationship.

## Spirituality and Religiosity: similarities and differences

What do we mean by the spiritual dimension of life? The term “spiritual” risks being understood in a partial or pre-judicial way. The pre-understanding that accompanies this word may lead to relegate it to religious, confessional contexts: it is thought that the spiritual dimension is specific to those who have an explicit faith and live a religious practice. Conversely, to bring this dimension to its broader meaning and recognize that spirituality belongs to every human being we may say that spirituality is a dimension of life and as such it must be recognized when considering human health.

There is much controversy and disagreement regarding definitions of spirituality and religiosity. While some research refers to the concepts of spirituality/religiosity (S/R) interchangeably there are differences between the two concepts. Much of the confusion stems from the fact that spirituality and religiosity are often, but not always, related, leading to considering diverse concepts such as God, meaning, mystical, the sacred, transcendence, and faith overlapping [13]. Many researchers adopt the option of combining these concepts in S/R (or R/S) to refer to these intertwined constructs, but the divergence in the use of these notions contribute to the heterogeneity of the approaches and results of studies dedicated to this area [3]. While the term S/R is useful when discussing generalities regarding the associations with health outcomes, S/R encompasses various dissimilar dimensions. Moreover, each of these dimensions may exert specific and unique effects on health. Hence, understanding the results of specific studies depends to a great extent on the particular dimension(s) of S/R studied.

One of the authors with large experience in research on this topic is Harold G. Koenig, who has defined religion and spirituality as follows:

“[Religion] Involves beliefs, practices, and rituals related to the transcendent, where the transcendent is God, Allah, HaShem, or a Higher Power in Western religious traditions, or to Brahman, manifestations of Brahman, Buddha, Dao, or ultimate truth/reality in Eastern traditions. This often involves the mystical or supernatural. Religions usually have specific beliefs about life after death and rules about conduct within a social group. Religion is a multidimensional construct that includes beliefs, behaviors, rituals, and ceremonies that may be held or practiced in private or public settings, but are in some way derived from established traditions that developed over time within a community. Religion is also an organized system of beliefs, practices, and symbols designed (a) to facilitate closeness to the transcendent, and (b) to foster an understanding of one’s relationship and responsibility to others in living together in a community” [14].

“Spirituality is distinguished from all other things—humanism, values, morals, and mental health—by its connection to that which is sacred, the transcendent. The transcendent is that which is outside of the self, and yet also within the self—and in Western traditions is called God, Allah, HaShem, or a Higher Power, and in Eastern traditions may be called Brahman, manifestations of Brahman, Buddha, Dao, or ultimate truth/reality. Spirituality is intimately connected to the supernatural, the mystical, and to organized religion, although also extends beyond organized religion (and begins before it). Spirituality includes both a search for the transcendent and the discovery of the transcendent and so involves traveling along the path that leads from nonconsideration to questioning to either staunch nonbelief or belief, and if belief, then ultimately to devotion and finally, surrender. Thus, our definition of spirituality is very similar to religion and there is clearly overlap” [14].

Thus, according to Koenig [15], on one hand, religion is a system of beliefs and practices observed by a group of people that are based on rituals or a set of Scriptures and teachings “that recognize, worship, commune with the Sacred, the Divine, God”. On the other hand, spirituality is a quality of the nature of the spirit, a factor pertinent to every human being. For Puchalski [16], one of the pioneers of the movement to integrate spirituality into health care, spirituality is each person's inherent search for the ultimate meaning and purpose of life. This meaning can be found in religion, but can often be broader than that, including a relationship with a divine figure or with transcendence, relationships with others, as well as the spirituality found in nature, art and rational thinking. Pargament et al. [17] define spirituality as the search for meaning, the belief in a higher power, and finding happiness and joy in everyday life.

Thus, spirituality is a domain (inside or outside a religion) that can be present in any human experience—such as in its values, morals, ethics, love, compassion, art, connection, inner peace, hope, energy, joy, strength, support, friendship, solidarity, humanism, comfort—and, especially, in the meaning and purpose of life. All of these aspects can influence how patients and healthcare professionals perceive health and disease and how they interact with each other. In this context, spirituality is one of the indicators of the notion of health.

## Evidence of the association between spirituality/religiosity and longevity

Tyler VanderWeele, a renowned author on spirituality and religiosity in relation to health outcomes, argues that the regular search for causes of mortality and morbidity or risk factors such as smoking, obesity or drug overdose is valuable but incomplete because do not consider important concerns that are important for people’s daily lives. He emphasizes that in the definition of health given over 70 years ago by the World Health Organization as “a state of complete physical, mental and social well-being and not merely the absence of disease and infirmity” also other components of well-being besides physical health are embraced [18, 19].

Other fields investigating determinants of human well-being including psychology, economics and sociology generally exclude physical health as a fundamental parameter of well-being. What current explorations seek is to contemplate all the dimensions that can ultimately contribute holistically to health including S/R variables [13, 19]. For the purpose of these investigations, different related dimensions and variables have been taken into account (Table 1) and some evaluation tools have been developed (Table 2).

**Table 1:** The 7 × 7 dimensions of spiritual assessment developed by George Fitchett et al. [20]

Holistic assessment	Spiritual assessment components
Medical	Belief and Meaning
Psychological	Vocation and obligations
Family systems	Experience and emotions
Psychosocial	Courage and growth
Ethnic, racial or cultural	Ritual and practice
Social issues	Community
Spiritual	Authority and guidance

**Table 2:** Examples of assessment tools to evaluate S/R dimensions

Assessment tool	Components	References
FICA spiritual assessment	Faith or beliefs Importance & influence Community Address or application	Puchalski CM, 2014 [21]
HOPE	Sources of Hope Organized religion Personal spirituality and practices Effects on medical care issues and end of life issues	Snyder CR, Lopez SJ, 2009 [22] Anandarajah G, Hight E, 2001 [23]
SPIRITual history	Spiritual belief system Personal spirituality Integration with spiritual community Ritualized practices and restrictions Implications for medical care Terminal events planning	Maugans TA, 1996 [24]
Flourishing measures and questions	Happiness Mental and physical health Meaning and purpose Character and virtue Close social relationships Financial stability	VanderWeele TJ, 2017 [18]
Spiritual distress assessment tool	Meaning Transcendence Values Psychosocial Identity	• Monod SM, et al. 2010 [25]

In recent years, evidence has accumulated on the impact of spirituality and religiosity on various health outcomes that are related to longevity; this includes associations with decreased risk of mortality [4–8, 15, 26–28], cardiovascular disease (CVD) [29–33], cancer [34–36], suicide [37], and also cognitive decline [38] and healthy aging that leads to healthy longevity.

### Mortality

One of the outcomes with the greatest number of investigations in relation to S/R is mortality, both total and cause-specific. In an extensive review [15], Koenig indicated that until 2010 at least 121 studies examined this relationship, most of them were prospective cohort studies controlling for multiple confounders. Of those studies, 68% found that greater S/R significantly predicted greater longevity, while 5% reported the opposite. Considering 63 studies with the most rigorous methodology, 75% reported that S/R predicted greater longevity, while, again, 5% reported the contrary. Two meta-analyses [26, 27] and a systematic review [28] confirmed these results with effects particularly strong for frequency of religious services attendance. Survival among frequent attendees of religious services was increased on average by 30%, 37% and 43%, respectively [26–28], which is surprising because these results are similar to or better than many medical interventions.

More recently, analyses of data from the Nurses’ Health Study (*n* = 74,534; followed from 1992 to 2012) reported that after multivariable adjustments for relevant factors, attending a religious service ≥ once/week was associated with 33% lower all-cause mortality, 27% lower cardiovascular mortality, and 21% lower cancer mortality compared to women who had never attended religious services. [8]. Thus, S/R may be an underappreciated resource that physicians could explore with their patients. Other studies indicate that psychological well-being domains closely linked to spirituality may contribute to shape physical health. For example, two meta-analyses have shown that purpose in life [4] and life satisfaction [5] were associated with reduced mortality risk. Contrariwise, social isolation and loneliness were related with increased mortality risk [6]. A recent prospective study examined the association of religious service attendance and deaths from despair (related to drugs, alcohol, and suicide) reporting that health professionals who attended religious services ≥ once/week had a 68% lower risk of death from despair compared with those who never attended religious services, in the fully adjusted statistical models [7].

### Cardiovascular disease

Koenig found that among 19 studies examining the association between S/R and coronary heart disease (CHD), 63% reported a significant inverse relationship, and one study reported a direct association [15]. Considering only 13 studies with most rigorous methodology, 69% found a significant inverse association between S/R and CHD.

A recent cross-sectional study [29] among 2967 participants of the Jackson Heart Study analyzed the association of S/R (religious attendance, private prayer, religious coping) with the American Heart Association (AHA) Life’s Simple 7 indicators (LS7) (not smoking, healthy weight, eating healthy, being physically active, blood pressure [BP], cholesterol and blood sugar), The results showed that higher religious attendance was associated with increased likelihood of achieving intermediate/ideal levels of physical activity, diet, smoking, BP, and LS7 composite score. Private prayer was associated with increased odds of achieving intermediate/ideal levels for diet and smoking. Religious coping was associated with increased odds of achieving intermediate/ideal levels of physical activity, diet, smoking, and LS7 composite score. These results reinforce the notion that S/R effects on lifestyle interventions may help decrease overall CVD risk among African–Americans.

#### Hypertension

According to Koenig’s review [15], 57% of the 63 studies examining the association between S/R and BP reported lower values in those most adherent to S/R while 11% reported significant higher BP levels, with similar results when considering only high-quality studies [15]. To help explain the results reporting higher levels of BP in those more adherent to S/R practices, Koenig argued that most of the population included were African–Americans, who are the most religious ethnic group in the society and also more likely to have high BP values. There are other factors that can be confusing when evaluating parameters dependent on multiple determinants as BP in dissimilar populations. To overcome the fact that most of the studies showing protection of S/R with respect to hypertension (HTN) have been conducted in white populations with limited S/R measures (i.e., religious service attendance), a recent cross-sectional study [30] evaluated four racial/ethnic groups and diverse S/R variables, including individual prayer, group prayer, nontheistic daily spiritual experiences, yoga, gratitude, positive and negative religious coping. This study found different patterns of associations depending on gender and ethnicity. Among women: (1) religious attendance was associated with lower HTN among Black and white women; (2) gratitude was linked to lower HTN among Hispanic/Latino, South Asian, and white women; (3) individual prayer was associated with higher HTN prevalence among Hispanic/Latino and white women; (4) yoga was associated with higher HTN among South Asian women; and (5) negative religious coping was linked to higher HTN among Black women. Among men: significant results were only found among Hispanic/Latino men. Religious attendance and individual prayer were associated with higher HTN, while group prayer and negative religious coping were associated with lower HTN. These results reflect the multifaceted nature of the S/R construct with diverse manifestations by race/ethnicity and gender, which encourages the avoidance of considering a single approach to these complex outcomes and dimensions.

#### Heart failure

Heart failure (HF) is currently among the most prevalent chronic diseases with estimates in the US of over 6.5 million people living with HF [39]. Clinical manifestations of HF comprise disturbing symptoms, instability in fluid management and sodium intake, and need of frequent monitoring and hospitalizations [39], which worsen quality of life and is frequently associated with depressive symptoms [40]. There is evidence that factors related to S/R may be relevant in patients with HF [31], especially when the disease exacerbates over time [32]. We have recently reported the results of a systematic review of available literature aiming to evaluate the importance of S/R in patients with HF [33]. Among 810 non-duplicate records, we screened the full texts of 25 works. After relevant exclusions, 7 studies (3 observational and 4 interventional) comprising 1234 HF patients followed-up for a median of 3 months were reviewed. Despite the extreme heterogeneity of the populations included, of the definitions of S/R, and of the interventions in the few studies that included them, all the studies reported some positive associations with S/R. The intervention studies showed improvements in quality-of-life (QoL), in some cardiovascular outcomes, or decreased mortality. Regrettably, S/R are aspects that are not generally considered in the usual clinical practice and can potentially contribute to improving the conditions of chronically ill patients such as those with HF.

### Cognitive decline

By reducing stress and depression through enhanced effective coping, S/R may favor positive effects on cognitive performance. Among 21 studies published before 2010 examining the associations between S/R aspects and cognitive function in healthy persons and in dementia patients, 48% reported significant associations with better cognitive performance and 14% found the opposite [15]. In a recent systematic review [38] including 17 eligible studies, 82% reported positive associations between S/R and cognitive performance. Thus, public health practitioners should not overlook the benefits of enabling S/R practices among religious adults in the prevention of cognitive decline.

### Cancer

From 29 studies evaluating the association of S/R and incident cancer or outcomes in patients with cancer, including mortality, 55% found that those with more S/R practices had a lower risk of developing cancer or a better prognosis when already diagnosed with it vs. 7% reporting association with worse prognosis [15]. The favorable results may be partially explained by healthier behaviors (e.g., less smoking, alcohol abuse, more adherence to therapies) and/or a lower stress level and higher social support. Similarly, several investigations have correlated measures of S/R with better QoL and/or psychosocial functioning in the context of various types of cancer [34], particularly in patients with advanced cancer.

There is growing interest on the role of S/R practices in cancer prevention. A recent systematic review [35] evaluated the influence of religious denominations, the importance of religion in one’s life, and religious practices such as church attendance, on the utilization of cancer screenings. Most of the 27 studies included reported a positive association of religious attendance with cancer screening utilization and mixed evidence concerning religious denomination and religiosity. Some available meta-analyses on the study of S/R and health among cancer patients affirm that S/R is significantly though modestly associated with patient-reported mental, physical, and social health with some favorable but also sometimes poorer outcomes [36]. Nevertheless, the available data should encourage clinicians to considered S/R for the holistic patient-centered care.

### Healthy aging

With the increasing aging populations, interest has grown globally in identifying factors that may contribute to healthy aging and longevity. Key aspects on the role that S/R may play on the promotion of healthy aging include the increased social support [41], the associations with improved QoL [42], decreased mortality and reduction of some chronic conditions discussed above, psychological and mental health and resilience [43, 44], purpose in life [45], improved cognitive function [38], and better management of end-of-life and death issues [46]. Yet, it is essential to bear in mind that there are significant differences in terms of sex, ethnicity, cultural background, and characteristics of family nuclei and communities regarding the interpretation of the role that S/R can play in healthy aging. This may generate profound difficulties in the application of S/R interventions that can help in the promotion of healthy longevity.

## Potential mechanism to explain the association between spirituality/religiosity and longevity

**Fig. 1:**
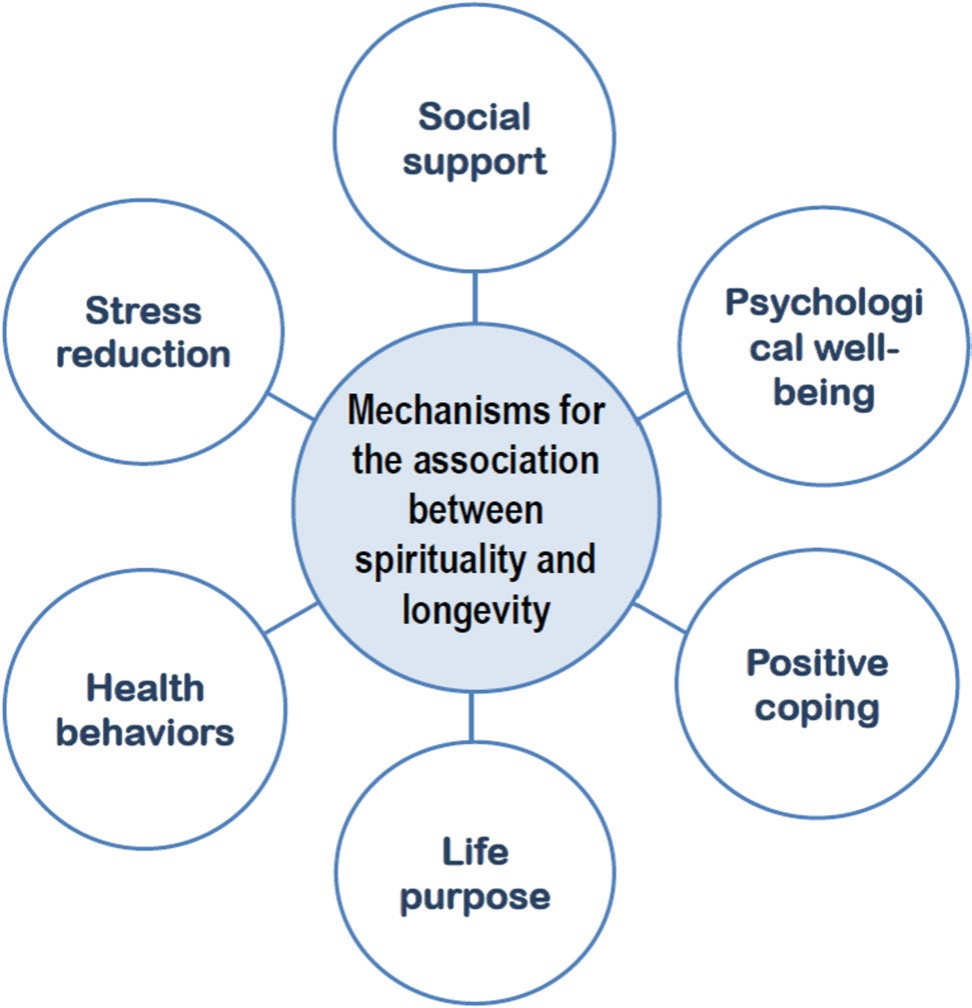
Main mechanisms helping to explain the association between S/R practices and longevity

**Fig. 2:**
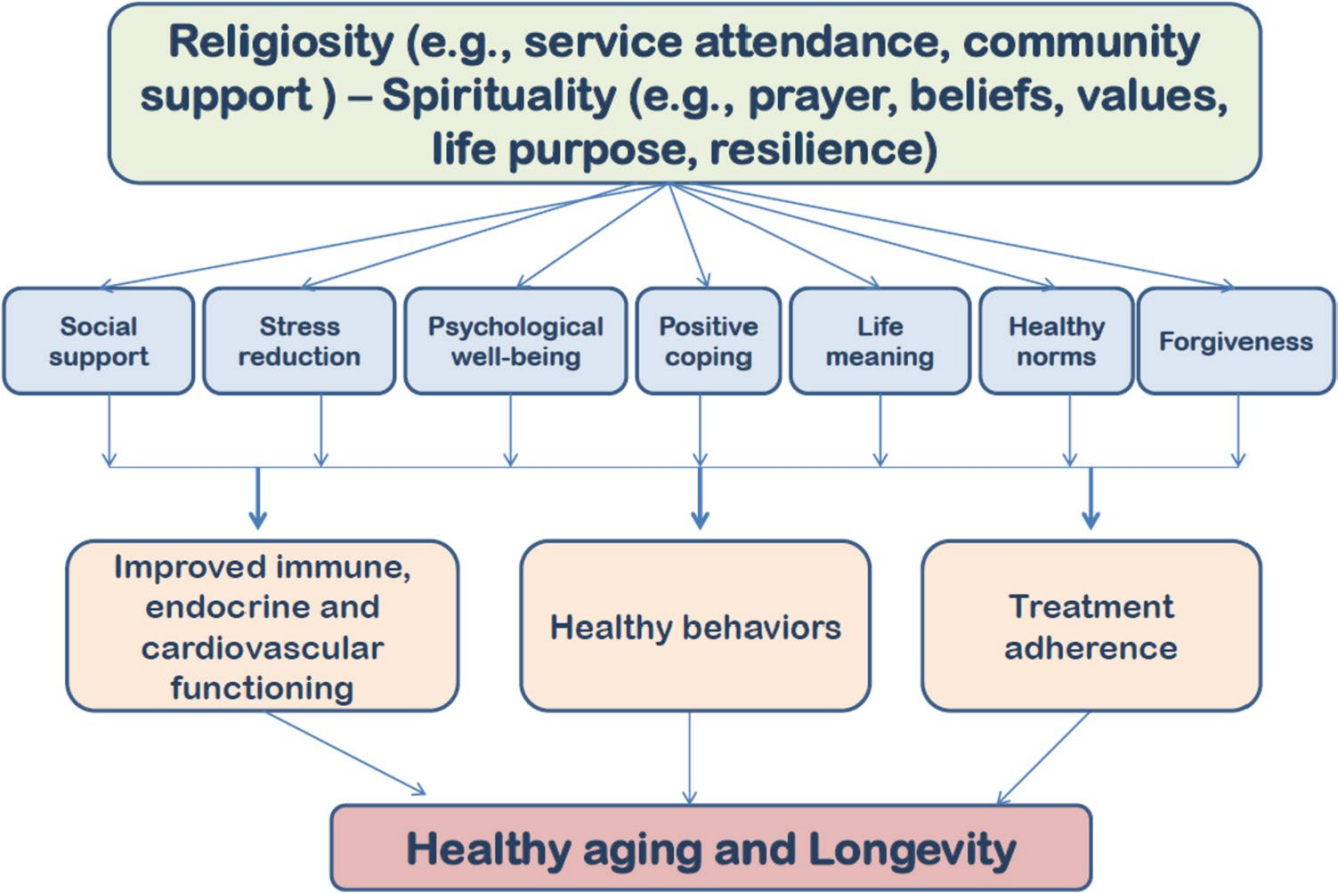
Suggested pathways to explain the association between S/R practices and longevity

Several mechanisms and pathways are suggested to help explain how S/R may exert beneficial effects on longevity and well-being shown in Figs. 1 and 2 as will be discussed below.

### Stress reduction

Engaging in spiritual practices, such as meditation, prayer, or mindfulness, may help reduce stress and promote relaxation. Chronic stress has been linked to various health issues, including CVD, HTN, and weakened immune function through several pathways [47]. Persons with S/R regular practices have shown better abilities handling emotion in stressful situations [48]. By managing stress and finding effective stress-reducing techniques people may improve their overall health and potentially influence their life expectancy.

Some studies have reported that a meditation program is able not only to improve sleeping quality, but also to increase brain plasticity, strengthening the ability to process and store data [49, 50]. Meditation techniques have been shown to inhibit sympathetic system activity and activate the parasympathetic system [51], which may explain the reduction of stress. Stimulation of the parasympathetic system inhibits inflammation, a mechanism associated with many age-associated chronic diseases. The release of acetylcholine from the vagus nerve suppresses gene expression and the secretion of inflammatory proteins by binding to specific inhibitory receptors on macrophages [52]. Achievement of introspection skills via meditation has been associated with lower levels of anxiety, and depression, as well as higher levels of self-esteem and satisfaction [53]. People who meditate on a regular basis as an S/R practice tend to obtain a greater capacity for introspection, self-awareness, self-control, and management of emotions, which reflect the optimal integration of autonomic, affective and cognitive processes.

### Social support

Spiritual practices often involve being part of a community or religious group, which can provide a strong social support network. Epidemiological studies have shown that social relationships are crucial determinants of health and longevity [54]. Socially isolated people are physically and psychologically less healthy, and have a higher mortality risk compared with persons with regular social contacts. For example, unmarried people have a higher mortality rate than married counterparts and the risk of death doubles in men and triples in women within the first month after the partner's death [55]. Recent analyses of data from the Health and Retirement Study reported that both loneliness and social isolation were independently associated with several physical health outcomes and behaviors after adjusting for a wide range of covariates [41]. Hence, loneliness and social isolation constitute distinct targets for interventions aimed at improving population health and well-being. Participation in an S/R community not only provides supportive social connections and opportunities for altruism (through volunteering or other faith-based altruistic activities), but also increases the flow of health information that may improve disease screening and promote health maintenance. Analyses from the Nurses' Health Study reported that women who were more socially integrated were more likely to be healthy agers (no history of major chronic disease diagnosis, no self-reported impairment in memory, and no major impairments in physical function or mental health) [56].

The social support networks of those who belong to a religious community are larger than those who do not attend religious services regularly, and may be qualitatively different from other non-religious social support [57]. However, religious social interactions are not always positive or related to better health. A longitudinal study of a national sample of African–Americans reported that positive religious social support was associated with higher consumption of fruits and vegetables, and lower depressive symptoms and heavy drinking over time, but negative religious social interactions predicted more alcohol consumption, increased depressive symptoms, and decreased emotional functioning [58, 59].

### Positive coping mechanisms

Spiritual beliefs and practices can offer a framework for finding meaning and purpose in life, which may help understanding life's difficulties and cope with challenging situations. These positive coping mechanisms may help individuals deal with stress, trauma, or loss, which could, in turn, have a positive impact on their health and well-being. S/R may be related to health through optimism, or the degree to which one has positive expectations for the future. Over 80% of studies published prior to 2010 reported a significant positive relationship between optimism and S/R [15]. Positive perception and optimism have been associated with physical health [60, 61] and are frequently related with higher levels of S/R [62]. Religions explicitly promote positive emotions such as gratitude, compassion, and hope, all of which have been proposed to lead to greater physical and psychological well-being [18, 19]. People frequently turn to religion in their coping efforts. Pargament et al. have extensively studied religious coping types, classifying them as positive and negative [63]. Positive religious coping comprises making benevolent religious reassessments, using a collaborative approach with God in facing problems, seeking spiritual support, and seeking support from members of one’s religious community. Persons with high S/R practices are more inclined to use religious coping, which is often related to better psychological adjustments [17, 64].

### Health behaviors

Some spirituals beliefs and practices promote healthier behaviors (i.e., less alcohol and drug use, less cigarette smoking, more physical activity and exercise, better diet, and safer sexual practices). These lifestyle choices can contribute to better health and potentially lead to increased life expectancy.

Several studies confirm the relationship of S/R practices and healthy behaviors, for example, the recent study mentioned above in which African–Americans with greater religious attendance were more likely to achieving intermediate/ideal levels of physical activity, diet, smoking, BP, and LS7 composite AHA score [29]. Interestingly, the association of religiosity and healthy or unhealthy behaviors may depend on the type of spiritual beliefs. For example, a positive belief (e.g., that the person works in partnership with God to stay healthy) was associated with a higher fruit consumption while a passive spiritual believe (e.g., that a higher power controls the person’s health with no need of engaging in healthy behaviors) was associated with lower vegetable consumption and higher alcohol drinking [58]. Epidemiological evidence has reported that healthy behaviors partially mediate the effects of religious service attendance on physical health and mortality [65]. The influence of particular religions, or denominations, on health behavior and outcomes has been studied, but rarely as a primary focus [66].

Some religious communities and traditions clearly endorse health behaviors or vetoes that may influence health, such as prohibiting alcohol and other toxic substances or advising a vegetarian diet and banning sexual activity outside of marriage [67]. Other aspects of S/R have been linked to health behaviors. For example, in a large sample of African–Americans with diabetes, S/R beliefs and religious social support were associated with adhering to a healthier diet and better adherence to foot care [68].

However, religion may not only exert positive effects on healthy behavior but the opposite has also been observed. A study conducted in a large randomly selected community sample found that the Religious Involvement Index (church attendance, importance of religion, and religion as a source of comfort) had a small but positive correlation with higher obesity rate and more chronic disease and a weak correlation with both healthy and unhealthy eating behaviors [69]. Likewise, in a study of African–Americans, despite having more education, those from religious communities were more obese and had more diabetes and hyperlipidemia compared with non-religious African–Americans. However, participants from religious communities had higher rates of treatment and control of most cardiovascular risk factors (i.e., treated hyperlipidemia, controlled diabetes, controlled HTN, more physical activity, and less smoking) [70]. These results support the notion that the relationship between S/R and healthy behaviors is complex.

### Psychological well-being

Spirituality can be a source of comfort, hope, and inner peace, fostering a positive outlook on life that may lead to improved emotional and psychological well-being. Maintaining good mental health and a positive mindset have been linked to improved overall health and may indirectly influence longevity. In fact, S/R, by enabling coping and negative events overcoming with meaning and purpose, has been associated with better mental health (lower rates of depression, less anxiety, less stress, greater well-being and positive emotions). A study aiming to develop empirically based S/R typologies and their relationship with health and well-being analyzed data from 1431 adults. Typologies of S/R were derived based on religious service attendance, prayer, positive religious coping, and daily spiritual experiences. In multivariate statistical analyses, four clusters were identified: highly religious, moderately religious, somewhat religious, and minimally religious or non-religious. The highly religious class was most likely to be happy and satisfied with finances and least likely to be psychologically distressed [64].

A recent review [43] examined the scientific evidence on the relationship between S/R and both physical and mental health. The authors found solid evidence for reduced depression, suicidality, and substance use, but mixed or scarce results on other diagnoses, such as post-traumatic stress disorder, psychosis, and anxiety. They suggest that S/R effects on mental health are likely bidirectional, and the manner in which religious beliefs are used to cope with distress (i.e., negative and positive), may affect mental health outcomes. A recent study [71] examined the relationship between S/R and positive mental health and mental illness (i.e., psychological distress) and the potential moderating role of age using data from the 2012 Canadian Community Health Survey-Mental Health (CCHS-MH). Although S/R was associated with positive mental health among all participants, the association was stronger for older adults. These findings highlight the importance of S/R in positive mental health across the adult lifespan.

### Improved immunological and endocrine functioning

By promoting overall mental well-being, S/R practices may directly influence several biological systems, including the sympathetic nervous, endocrine, and immune systems [72]. Acute and chronic dysregulation of the stress system at different levels has been implicated as a major pathway and link to numerous behavioral (e.g., anxiety, depression, eating disorders, post-traumatic stress disorder, sleep disorders, etc.) and somatic disorders (e.g., chronic pain and fatigue syndromes, obesity, metabolic syndrome, chronic inflammation, type 2 diabetes, HTN, atherosclerosis, and CVD) [47]. A recent cross-sectional study analyzed data from 4,734 community-dwelling participants of the US Health and Retirement Study regarding the relationship between chronic stress, inflammation, and religiosity (organizational, non-organizational, and intrinsic religiosity), controlling for relevant covariates. This study found that intrinsic religiosity moderated the relationship between chronic stress and inflammation suggesting that persons with stronger religious commitment/motivation may better cope with stress [73].

Before 2010, Koenig et al. identified 27 studies evaluating the relationships between S/R and immune functions and found that 56% reported positive relationships or positive effects in response to an S/R intervention, and only one study (4%) found a negative effect. Considering only 14 studies with the highest quality ratings, 71% reported significant positive associations or increased immune functions in response to an S/R intervention [15].

A continuously growing area of interest and application in medicine is that related to mindfulness-based programs (MBP). Following the first studies by Kavat-Zinn et al. involving small groups and reporting the effectiveness of MBP in reducing symptoms of anxiety and panic disorders [53], various investigations have shown associations between mindfulness and various indicators of psychological health, including increased subjective well-being, reduced psychological symptoms and emotional reactivity, and improved behavioral regulation [74]. There are also studies showing the positive effects of meditation on objective measurements such as reduction of blood cortisol levels in the short and long term, as reported in a recent meta-analysis [75] including any type of meditation intervention but mostly concerning mindfulness. In 10 randomized controlled trials among 395 participants there was a significant, medium-sized effect of meditation intervention on changes in blood cortisol levels. Because there is an explosion of studies that use MBP interventions in different health outcomes, it is important to be aware that “mindfulness” is a generic concept and that more detailed elucidations on what is and what is not an MBP are necessary to delineate more rigorous investigations in the future [76].

## Conclusions

With the continuous growth of population aging, multidisciplinary and multidimensional strategies that can help to manage the complex situations of these populations are needed. Interventions for this purpose should consider the promotion of the physical, psychological, behavioral, and social health of older adults who are in great need on all these fronts. S/R comprise areas that have attracted and continue to raise interest in health research. Numerous epidemiological studies have shown how higher levels of S/R in its different expressions are associated with a lower mortality rate, even after adjustments for relevant confounders. Other positive results have been reported in cardiovascular health and in well-being of cancer and psychiatric patients, but there are also negative and neutral results. Factors that may help explain the heterogeneity of the results and that can be challenging are the diverse conceptualization of S/R (from membership in organized religions and participation in their activities to individual practices, such as prayer, meditation or considering oneself a religious person) and the dissimilar methods used for S/R assessment. In addition, the difficulty of implementing programs in which there is convincing transferring of the results in effective spiritual care interventions in the real-world confirming the concept of transitional epidemiology [3]. This may contribute to the resistance and struggle in the acceptance of this type of interventions, despite the growing medical literature dedicated to these arguments.

Because S/R are dimensions of great value for patients, overlooking them can leave patients with feelings of neglect and lack of connection with the health system and with the clinicians in charge of their care. Integrating S/R assessment and intervention programs into clinical care can help each person obtain better and complete well-being and also allowing clinicians to achieve the highest standards of health with holistic, person-centered care, always remembering that S/R is a deeply personal aspect of life and that its effects on health can vary greatly from person to person. S/R should not be viewed solely as a way to prolong longevity. People approach spirituality seeking personal growth, emotional support, and purpose in life.

While there might be some correlations between S/R and health outcomes, they do not necessarily imply causation and not all spiritual beliefs or practices are beneficial for everyone: there is no “one-size-fits-all” approach to spirituality. Ultimately, the decision to embrace spirituality should be driven by personal beliefs, values, and experiences rather than an expectation of prolonging life.
